# Opportunities for Nanomaterials in Stretchable and Free‐Form Displays

**DOI:** 10.1002/smsc.202300143

**Published:** 2024-01-29

**Authors:** Yeageun Lee, Weilin Guan, Ezekiel Y. Hsieh, SungWoo Nam

**Affiliations:** ^1^ Department of Mechanical and Aerospace Engineering University of California, Irvine Irvine CA 92697 USA; ^2^ Department of Mechanical Science and Engineering University of Illinois at Urbana-Champaign Urbana IL 61801 USA; ^3^ Department of Materials Science and Engineering University of California, Irvine Irvine CA 92697 USA

**Keywords:** free-form displays, nanomaterials, stretchable displays, wearable displays

## Abstract

Stretchable and free‐form displays receive significant attention as they hold immense potential for revolutionizing future display technologies. These displays are designed to conform to irregular surfaces and endure mechanical strains, making them well suited for applications in wearable electronics, biomedical devices, and interactive displays. Traditional light‐emitting devices typically employ brittle inorganic and metallic materials, which are not conducive to stretchability. However, replacing these nonflexible components with flexible/stretchable nanomaterials, soft organic materials, or their composites improves the overall flexibility and stretchability of devices. In this review, the roles and opportunities of nanomaterials, such as thin films, 1D nanofibrous materials, and micro/nanoparticles, are highlighted for enhancing the stretchability and overall performance of various types of light‐emitting devices. By leveraging the unique mechanical and electrical properties of nanomaterials, various efforts emerge to push the boundaries of stretchable display technologies and further realize their full potential for diverse applications.

## Graphical abstract

The roles and opportunities of nanomaterials in enhancing the stretchability and overall performance of stretchable light‐emitting devices are discussed. Various nanomaterials utilized in intrinsically stretchable displays are comprehensively reviewed. Different structural strategies employed in stretchable displays and the role of nanomaterials within these devices are also presented.

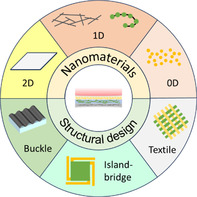

## 1 Introduction

Stretchable and free‐form displays have become essential for the advancement of future electronic devices, encompassing a wide range of applications such as expandable displays, interactive displays, wearable technologies, internet of things (IoT) devices, biomedical applications, and more.^[^ ^1^, ^2^ ^]^ The extreme deformability of stretchable displays opens the door to the next generation of advanced user‐interactive display technology, where the display form factor can adapt to the content being shown, extending the concept of Braille display systems. These displays boast novel and unconventional form factors with 3D deformability, allowing them to conform to curvilinear shapes such as soft human skin. This additional degree of deformation freedom minimizes user discomfort when securely attached to the skin, facilitating the detection of physiological signals without motion artifacts caused by contact interface slippage resulting from elastic modulus mismatch. Such capabilities enable various biomedical and IoT applications, including attachable, implantable, and wearable devices for personalized real‐time health monitoring, prosthetics, soft robotics (enabling multifunctionality), smart clothing, and automotive features.

While flexible displays have made significant progress and gained market traction through the introduction of foldable or rollable devices,^[^ ^3^, ^4^, ^5^, ^6^, ^7^, ^8^, ^9^ ^]^ their current capabilities are limited to simple folding or rolling motions and fall short of meeting the requirements of advanced applications. To be applied to diverse future applications, displays must possess not only robustness and softness but also the ability to stretch and bend locally without compromising image quality or electrical/optical performance. Achieving stretchable displays requires seamless adaptation to convex and concave surfaces, withstanding elongation caused by pulling forces in the plane, and maintaining operational functionality with minimal degradation or failures under strains of at least 30%.^[^ ^2^, ^10^ ^]^

To achieve stretchability in displays, two major approaches have been pursued. The first approach involves the integration of intrinsically stretchable materials into each component of the light‐emitting devices, enabling the device to stretch as a whole (i.e., intrinsically stretchable). In this case, the traditional materials used in non‐stretchable displays are replaced by intrinsically stretchable materials for electrodes, light emission layers, and other functional layers such as hole‐transport and encapsulation layers. Consequently, polymer‐based organic light‐emitting diodes (OLEDs) have emerged as the dominant technology in this field. The second approach is focused on designing stretchable structures that allow non‐stretchable materials to be stretched through structural modifications (i.e., structurally stretchable). This approach involves the use of buckled materials, island–bridge structures, auxetic kirigami, and origami structures. The advantage of structurally stretchable designs is that they offer more flexibility in material selection. However, achieving a completely zero‐strain formation throughout the device during deformation is still impossible. Consequently, the development of strain‐endurable materials remains to be important, even though the required strain endurability is much lower compared to the intrinsically stretchable materials.

Nanomaterials, including nano‐thin films, nanofibrous materials, and quantum dots (QDs) have been actively utilized to realize stretchable displays. Nanomaterials offer the advantage of high strain tolerance by reducing the thickness or size of materials toward the atomically thin limit, thereby decreasing their mechanical stiffness.^[^ ^11^, ^12^, ^13^, ^14^ ^]^ Integrating these nanomaterials into the fabrication of electroluminescent devices and displays enables stretchability and softness beyond conventional light‐emitting devices.^[^ ^15^ ^]^ In addition, by reducing the thickness of material layers and positioning them closer to the neutral plane or zero‐strain position, the bending radius and strain in brittle materials such as the emissive layer can be minimized. Nanomaterials also exhibit superior strain tolerance when compared to their bulk counterparts, allowing them to withstand substantial deformation without compromising their electrical properties.^[^ ^16^ ^]^ This characteristic makes nanomaterial–elastomer mixtures highly desirable for the components of intrinsically stretchable displays, as they demonstrate excellent robustness under strain until the nanomaterial percolation is lost.^[^ ^17^, ^18^, ^19^, ^20^, ^21^ ^]^ Overall, nanomaterials provide an additional degree of freedom in the design of stretchable light‐emitting devices, enabling both intrinsic stretchability and structural modifications to be incorporated.

There have been several recent review reports about stretchable displays,^[^ ^1^, ^2^, ^10^, ^15^, ^16^, ^22^, ^23^ ^]^ but the roles and opportunities of nanomaterials in the development of stretchable displays were not discussed in depth. In this comprehensive review, we focus more specifically on nanomaterials and their potential contributions to stretchable and free‐form displays. First, we explore the application of 2D, 1D, and 0D nanomaterials in the development of intrinsically stretchable devices, highlighting their unique properties and functionalities. By categorizing nanomaterials based on their dimensions, we efficiently examine the merits of using different dimensional nanomaterials in intrinsically stretchable displays. Subsequently, we analyze the use of nanomaterials in structurally stretchable devices, showcasing their diverse applications and impact on display performance.

## 2 Intrinsically Stretchable Displays

The combination of intrinsic stretchability and flexibility ensures a high degree of deformation freedom, enabling truly free‐form devices. To realize intrinsically stretchable display devices, it is essential to incorporate stretchable components throughout the device, including electrodes, light emissive layers, electron/hole‐transport layers, encapsulation layers, and other functional layers. In addition, for vertical light‐emitting diodes (LEDs), with vertically stacked light‐emitting and functional layers between the top and bottom electrodes, it is crucial that the layers above the light‐emitting layer are transparent. Therefore, highly elastic and optically transparent polymers such as polyurethane (PU) and poly(dimethylsiloxane) (PDMS) have been widely employed as substrates or elastic matrices in which nanomaterials are embedded. Another commonly used material in intrinsically stretchable displays is poly(3,4‐ethylenedioxythiophene):polystyrene sulfonate (PEDOT:PSS), which serves as both a conducting polymer and a charge‐transport layer.^[^ ^22^ ^]^ These polymers, when combined with nanomaterials, serve as the building blocks for fabricating intrinsically stretchable display devices. By harnessing the electronic and optical properties of nanomaterials and leveraging the high stretchability of polymers, nanomaterial–polymer composites provide a versatile platform for tailoring the mechanical, electrical, and optical properties of display components. In this context, various types of nanomaterials play a crucial role. The 2D nanomaterials, such as nanoscale thin films and graphene, offer high flexibility due to their thin nature. The 1D nanomaterials, including nanowires (NW) and nanofibers, provide enhanced mechanical flexibility while maintaining their continuous network. The 0D nanomaterials, such as nanocrystals and QDs, exhibit exceptional optical properties and tunability.^[^ ^24^, ^25^, ^26^ ^]^ In the following section, we will explore the specific roles and contributions of these nanomaterials in intrinsically stretchable displays.

### 2.1 Thin Films and 2D Materials for Intrinsically Stretchable Displays

Nanoscale thin films offer unique advantages due to their ability to bend and buckle while maintaining their electrical properties, making them highly suitable for flexible electronics.^[^ ^22^ ^]^ Accordingly, the concept of flexible displays using thin‐film structures was introduced several decades ago.^[^ ^27^, ^28^, ^29^, ^30^, ^31^ ^]^ However, it is important to acknowledge that if the materials themselves are not inherently stretchable, thin films made from those materials will also have inherent limitations to their stretchability. Consequently, their use as major components in intrinsically stretchable displays has been relatively limited. Nonetheless, there have been research efforts to develop stretchable displays using thin‐film materials in the early development stages of stretchable display technology.^[^ ^32^, ^33^, ^34^, ^35^, ^36^, ^37^, ^38^ ^]^ In more recent studies, thin films have been employed to provide additional functionality or enhance the stability of certain device components.

**Figure 1:**
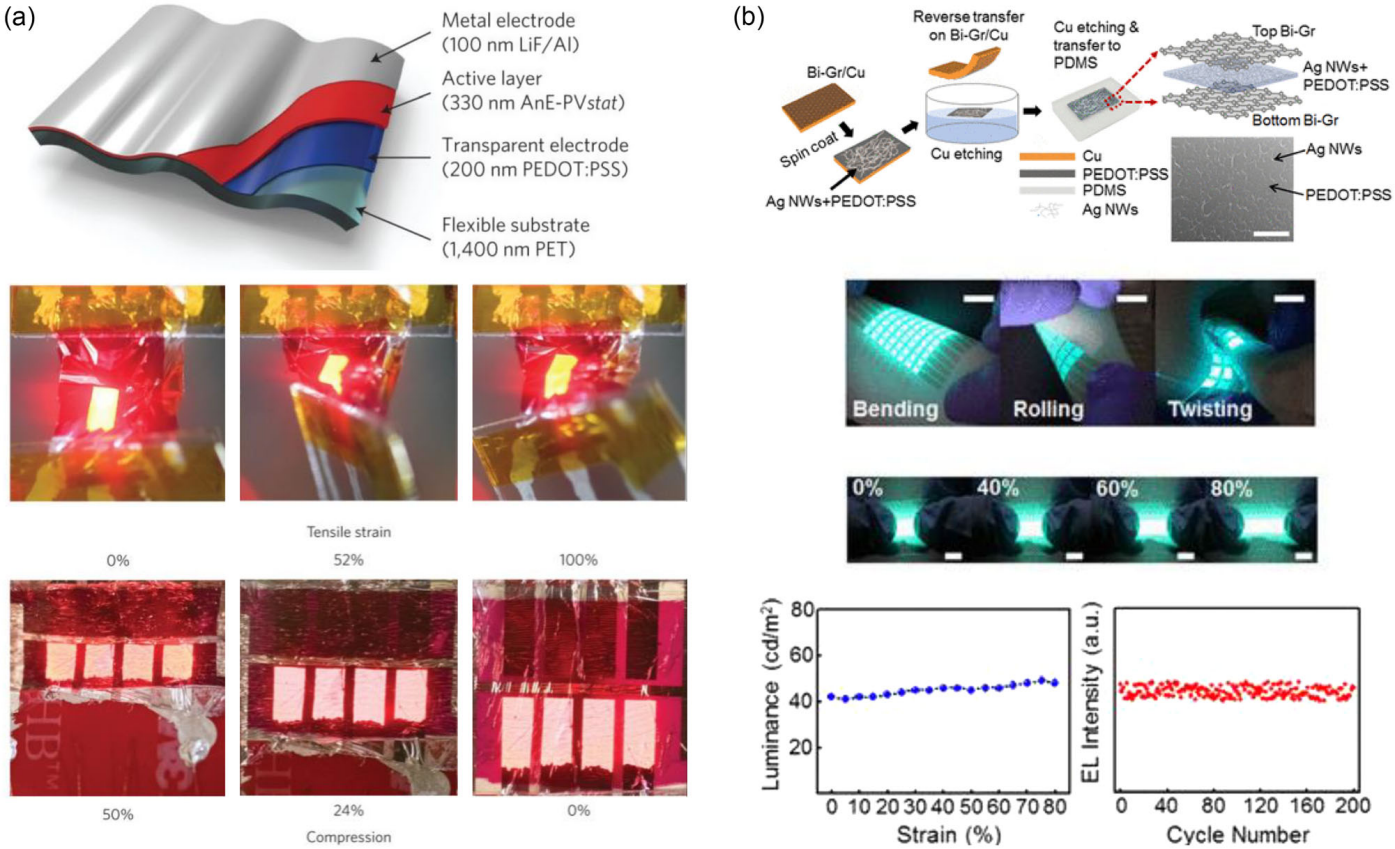
Schematic illustrations of intrinsically flexible or stretchable displays enabled by thin 2D film. a) Ultrathin polymer‐based organic light‐emitting diode (PLED) with nanoscale layers. The device operation during crumpling (as a free‐standing form) and stretching (as a wrinkled form adhered to a pre‐strained elastomer tape) were demonstrated. In the bottom panel, tensile strain indicates the applied strain to the wrinkled device, whereas the compression shows the corresponding strain at the PLED layer. Reproduced with permission.^[^ ^32^ ^]^ Copyright 2013, Springer Nature. b) Stretchable alternating‐current electroluminescent (ACEL) devices enabled by bilayer graphene (Bi‐Gr)/Ag NWs–poly(3,4‐ethylenedioxythiophene):polystyrene sulfonate (PEDOT:PSS)/Bi‐Gr sandwich electrodes. Luminance of the device at different strains and robust intensity under cyclic test are also displayed. Reproduced with permission.^[^ ^40^ ^]^ Copyright 2019, American Chemical Society.

Reducing device thickness plays a crucial role in shrinking the allowable bending radius of the devices, improving flexibility and enabling further device modifications to attain stretchability. For instance, White et al. reported polymer‐based organic LEDs (PLEDs) fabricated with an ultrathin thickness of 2 μm, as shown in **Figure** **1**a.^[^ ^32^ ^]^ The PLEDs consisted of a 200 nm‐thick conducting polymer (PEDOT:PSS) electrode, a 330 nm‐thick semiconducting polymer (anthracene‐containing poly(*p*‐phenylene‐ethynylene)‐*alt*‐poly(*p*‐phenylene‐vinylene) derivative with a statistical distribution of linear octyloxy and branched 2‐ethylhexyloxy side groups), and a 100 nm‐thick LiF/Al electrode, on a flexible poly(ethylene terephthalate) (PET) substrate with a thickness of 1400 nm. The device's ultrathin profile significantly reduced its bending radius to 10 μm, which makes it highly suitable for bendable or wearable displays. The PLEDs demonstrated reliable operation under free‐standing, compressing, bending, and twisting conditions. Moreover, the device was able to be stretched up to 100% strain on a pre‐strained substrate, as displayed in the bottom panel of Figure 1a. However, the large stretchability was only applicable after mounted on a pre‐strained substrate, which led to the wrinkling of the device, indicating structural modification. In another study by Yin et al., sub 200 nm thin OLEDs were developed on a photopolymer substrate.^[^ ^33^ ^]^ The device architecture included various organic and inorganic layers, such as an Ag anode layer, MoO_3_ anodic modification layer, *N*,*N*′‐diphenyl‐*N*,*N*′‐bis(1,1′‐biphenyl)‐4,4′‐diamine hole‐transporting layer, tris(2‐phenylpyridine)iridium(III) (Ir(ppy)_3_)‐doped *N*,*N*′‐dicarbazolyl‐3,5‐benzene‐emitting layer, 1,3,5‐tris(*N*‐phenyl‐benzimidazol‐2‐yl)benzene electron‐transporting layer, and Ca/Ag cathode layer. Each of these layers was engineered to have nano‐thin thicknesses ranging from 80 nm down to 3 nm, enabling the overall device to achieve mechanical flexibility. Yokota et al. also reported an ultraflexible organic photonic skin with PLEDs.^[^ ^34^ ^]^ A 1 μm‐thick parylene film coated with nanoscale polyimide (PI) planarization layer (500 nm) was used as a substrate material, and approximately 1.5 μm of alternating SiON and parylene layers were used as a cathode side passivation layer. Nanoscale indium tin oxide (ITO) anode, hole‐injection layer, interlayer, emissive layer, and NaF/Al cathode were positioned at the neutral plane between the parylene/PI substrate and the alternating SiON/parylene passivation layer. Reducing the thickness of the device layers to the nanoscale and placing them at the neutral plane provided remarkable flexibility. With a total thickness of 3 μm, the fabricated PLEDs displayed consistent electroluminescence even under a bending radius as small as 100 μm. In addition, owing to their thin form factor and low bending flexural rigidity, the fabricated PLEDs effectively demonstrated conformal contact with human skin. In combination with pre‐stretched acrylic tape–silicone rubber sheets, the PLEDs also demonstrated consistent functionality under 1000 cycles of 60% stretching strain. However, all these devices required pre‐strain to attain stretchability, resulting in structural modification of the thin films.

Despite its inherent limitations, thin metal films or nanoscale conductive films, such as graphene, have been investigated as composite materials with polymers for the development of intrinsically stretchable electrodes, without the need for structural modifications. These composite films aim to combine the high electrical conductivity of thin metal or graphene films with the stretchability and flexibility provided by the polymer matrix.^[^ ^35^, ^36^, ^37^, ^38^ ^]^ For instance, Yu et al. reported on the use of metal‐coated PU–PDMS composites for stretchable conductors.^[^ ^35^ ^]^ They coated a commercially available porous PU sponge with Cu, Au, or Cu–Ag bilayer films using electroless deposition. The resulting PU–metal samples were then embedded in PDMS to form metal‐coated porous PU microstructures in PDMS (PU–metal–PDMS). Among the three metal film options, the composite with 1.12 μm‐thick Cu–Ag bilayer coating (PU–CuAg–PDMS) exhibited the lowest sheet resistance, 1.48 Ω sq^−1^. The composite also demonstrated excellent conductivity retention under 1000 cycles of 40% compressive strain and 1000 cycles of 30% tensile strain. Even under 40% tensile strain, where the composite's resistance was approximately doubled compared to its unstretched state, it remained highly conductive. They further demonstrated the application of the PU–CuAg–PDMS composite as an interconnect material in a stretchable LED array, achieving 30% stretchability. However, the allowable bending radius of the composite was relatively large (4 mm) for highly flexible device applications. In addition, the optical images of the conducting layers indicated low transmittance, limiting their utility to only the bottom electrodes of devices or lateral LEDs, where the device layers are arranged in a lateral configuration on the substrate, allowing the light emissive layer to remain unobstructed by the top electrode. Moon et al. introduced Au nanosheets to build a conductive film on stretchable substrate.^[^ ^37^ ^]^ The nanosheets had a lateral size ranging from 20 to 50 μm and a thickness of approximately 20 nm. These nanosheets were initially suspended in 1‐butanol and assembled into a monolayer thin film on a water surface by pouring Au nanosheets/1‐butanol mixture in the water. After assembly, the thin Au films were transferred onto a target substrate. Depending on the number of transfer processes, the resulting Au films exhibited different thicknesses and conductivity levels. Each transfer step built a 30 nm‐thick film, and the resistivity gradually decreased with the sheet resistance of an eight‐time transferred film reaching 2–3 Ω sq^−1^ after annealing. When applied as stretchable conductors, the films demonstrated mechanical robustness under 2000 cycles of up to 100% tensile strain. Although these nanoscale Au films showed great promise as intrinsically stretchable conductors, similar to the PU–metal–PDMS composite by Yu et al.,^[^ ^35^ ^]^ the low optical transmittance of the films limited their applicability toward a variety of display applications. Liu et al. introduced graphene instead of thin metal films to develop optically transparent and mechanically stretchable electrodes.^[^ ^38^ ^]^ Graphene was synthesized on both sides of Cu foil during chemical vapor deposition. By protecting only one side of the graphene during the Cu wet‐etching process, the unprotected graphene formed a graphene/graphene scrolls structure. Through multiple transfers of this structure, a trilayer graphene/graphene scrolls structure on a stretchable elastomer (thermoplastic elastomer styrene–ethylene–butadiene–styrene) was created. The resulting stretchable conductor retained 65% of its original conductance at 100% strain, and the trilayer graphene/graphene scrolls exhibited nearly 90% transmittance across the visible light spectrum. The high transparency broadens the potential applications of the structure, though the conductivity was relatively low (>100 Ω sq^−1^) compared to other stretchable conductors.

Graphene and graphene oxide (GO) were also utilized to enhance the performance of intrinsically stretchable display devices.^[^ ^39^, ^40^, ^41^ ^]^ Shin et al. introduced 100 nm thin bilayer graphene (Bi‐Gr)/Ag nanowires (AgNWs)‐PEDOT:PSS/Bi‐Gr film as an electrode for stretchable alternating‐current electroluminescent (ACEL) devices, as shown in Figure 1b.^[^ ^40^ ^]^ In the absence of Bi‐Gr layers, the inhomogeneous network structure of AgNWs led to a nonuniform electric field throughout the AgNWs–PEDOT:PSS layer. As the material was stretched, the areas without AgNWs became larger, exacerbating the nonuniformity and resulting in unstable light emission throughout the device. The introduction of Bi‐Gr layers helped to reduce this nonuniformity while minimizing the loss of transmittance. An 8 × 8 ACEL device array with Bi‐Gr/AgNWs–PEDOT:PSS/Bi‐Gr electrodes was successfully demonstrated, exhibiting uniform intensities of electroluminescence under bending, rolling, twisting, and stretching strains of up to 80%, as depicted in Figure 1b. In another study, GO sheets with nanoscale thinness were utilized to further reduce the junction resistance of the AgNW network and enhance the chemical stability of AgNWs for stretchable PLED application.^[^ ^41^ ^]^ For AgNW networks on polymers with low coating density, preferable for display applications due to their high transmittance, soldering the AgNW junctions with GO improved conductivity of the networks more effectively than high‐temperature annealing at 180 °C for 30 min. Moreover, the AgNW networks covered by GO sheets exhibited remarkable stability, maintaining their conductivity for 8 days at 80 °C in ambient air, while the sheet resistance of the annealed AgNW network increased more than 4 times within 24 h. These findings highlight the potential of chemically stable thin‐film materials as functional layers of stretchable devices. In addition to graphene‐based 2D materials, thin stretchable elastomers such as liquid rubber compounds and PDMS have been employed as encapsulation layers to ensure device stability, particularly for stretchable OLED devices.^[^ ^42^, ^43^, ^44^, ^45^ ^]^ This is because well‐studied thin‐film inorganic encapsulation materials like silicon nitrides and aluminum oxides are unsuitable for use in intrinsically stretchable displays due to their extremely limited stretchability. However, it is worth noting that the organic encapsulation layers typically exhibit a high water vapor transmission rate (WVTR) in practical applications relative to their thickness.^[^ ^46^ ^]^ Increasing the thickness of the encapsulation layer to reduce WVTR would not be an appropriate solution, as it would impact the device's form factor and optical transparency. The field of stretchable encapsulation is still in its early stages of exploration, and the development of thin, highly stretchable, and transparent encapsulation layers remains a challenging area of research for the practical implementation of intrinsically stretchable displays.^[^ ^46^, ^47^, ^48^ ^]^

### 2.2 1D Nanofibrous Materials for Intrinsically Stretchable Displays

The 1D nanofibrous materials have emerged as a promising option for stretchable electronics, serving both as electrodes as well as active layer materials. In particular, as composite materials with elastomers, they are able to maintain their interconnected network structure under tensile strain until their percolation breaks. Randomly distributed 1D materials have a higher probability of forming percolated networks when they possess longer lengths. Moreover, longer lengths can ensure percolation even with a lower density of 1D materials and a reduced number of junctions between them. In the case of conducting materials, the resistance of the junctions plays a crucial role in determining the overall resistance of the percolation networks. Therefore, possessing long lengths or high length‐to‐diameter ratios is crucial for 1D material‐based conducting layers, as it contributes to both high electrical conductivity and high transmittance.^[^ ^49^, ^50^, ^51^, ^52^ ^]^

Carbon nanotubes (CNTs) exhibit high length‐to‐diameter ratio with excellent carrier mobility and exceptional mechanical strength.^[^ ^53^, ^54^, ^55^, ^56^ ^]^ As such, CNTs have attracted considerable attention in early stages for various flexible and stretchable electronic devices, including thin‐film transistors (TFTs), sensors, and conductors.^[^ ^53^, ^54^, ^57^, ^58^, ^59^, ^60^, ^61^, ^62^ ^]^ Sekitani et al. presented one of the first stretchable LEDs featuring a single‐walled CNT (SWCNT)‐based conductor.^[^ ^63^ ^]^ The SWCNTs, possessing a high aspect ratio (lengths exceeding 1 mm and diameters of 3 nm), were blended into an ionic liquid (1‐butyl‐3‐methylimidazolium bis(trifluoromethanesulfonyl)imide) and 4‐methly‐2‐pentanone. To ensure uniform distribution of the SWCNTs throughout the gel without shortening their lengths, the mixture was subjected to jet milling. The resulting bucky gel was then combined with a fluorinated copolymer (vinylidenefluoride–tetrafluoroethylene–hexafluoropropylene) to produce an SWCNT–rubber composite gel that functioned as elastic conductor. Depending on the wt% of the SWCNTs, the SWCNT‐based conductor exhibited up to 100 S cm^−1^ of conductivity and 120% of stretchability. A 16 × 16 OLED array was integrated with the SWCNT‐based conductor demonstrating 30%–50% stretchability while maintaining almost 90% of the luminance compared to that of the Cu‐wired OLED device. However, the OLED units themselves were not stretchable due to the metallic or inorganic electrodes, and only the SWCNT‐based interconnect parts situated between the OLED units were stretchable, indicating incomplete intrinsic stretchability.

**Figure 2:**
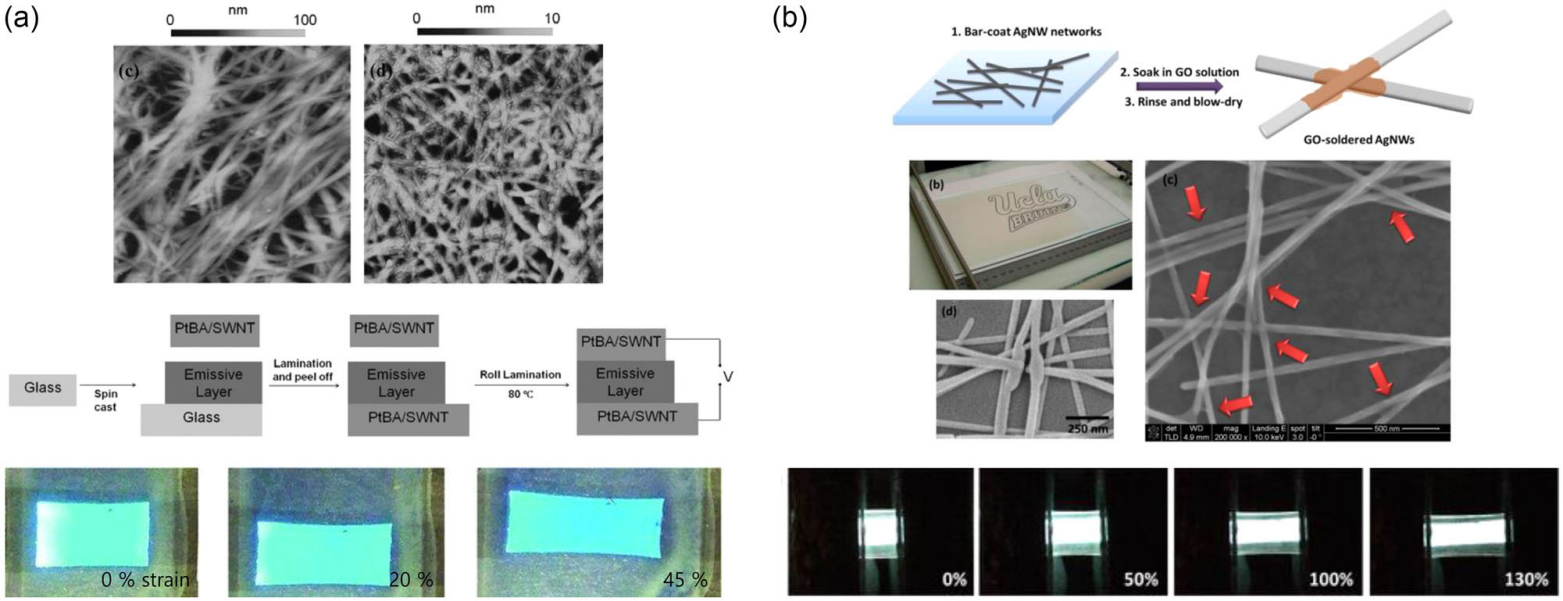
Schematic illustrations of intrinsically stretchable displays enabled by 1D nanofibrous materials. a) Atomic force microscopy (AFM) topographic images of single‐walled carbon nanotube (SWCNT), stretchable PLED structure with poly(tert‐butyl acrylate) (PtBA)/SWCNT electrodes, and device demonstration at different strain levels. Reproduced with permission.^[^ ^64^ ^]^ Copyright 2011, Wiley‐VCH. b) Schematic illustration and scanning electron microscopy (SEM) images of graphene oxide (GO)‐soldered AgNWs and 130% stretchable PLED with GO–AgNW/poly(urethane acrylate) (PUA) electrodes. Reproduced with permission.^[^ ^41^ ^]^ Copyright 2014, American Chemical Society.

To develop a fully intrinsically stretchable display, polymer‐based LED units were combined with SWCNT conductors. Yu et al. demonstrated a 45% stretchable LED with SWCNT–polymer composite electrodes.^[^ ^64^ ^]^ The SWCNT network was transferred onto a poly(tert‐butyl acrylate) (PtBA) substrate to create stretchable electrodes suitable for use as both anodes and cathodes in LEDs (**Figure** **2**a). Similar to the prior work by Sekitani et al.,^[^ ^63^ ^]^ thicker SWCNT networks exhibited higher conductivity but lower stretchability, likely due to an increased possibility of partial network breakage caused by more entangled SWCNTs. To create an intrinsically stretchable LED device, a luminescent polymer layer was inserted between the two SWCNT electrodes. The resulting devices could be stretched up to 45% without change in their light‐emitting properties as shown in Figure 2a. CNT‐based electrodes have also been applied to ACEL devices.^[^ ^65^, ^66^, ^67^ ^]^ Shi et al. developed a self‐healing and stretchable ACEL device with aligned CNT/PU composite electrodes.^[^ ^65^ ^]^ By applying pre‐strain during the device fabrication, the ACEL device achieved 400% stretchability with a ZnS phosphor‐based electroluminescent layer. More recently, Chang et al. introduced a self‐healing film based on SWCNT–PDMS for ACEL devices.^[^ ^66^ ^]^ This film was capable of withstanding uniaxial stretching up to 2500% strain without rupturing. Using this film, Chang et al. demonstrated multicolor ACEL devices that are intrinsically stretchable and can be stretched to 100% strain without requiring pre‐strain. Although SWCNT‐based electrodes are a promising material for the realization of highly stretchable display devices, the material cost and low conductivity of CNTs compared to metallic nanostructures limit their application.^[^ ^68^, ^69^, ^70^ ^]^

Metal NWs have also been proposed as an alternative to CNTs due to their excellent conductivity, but maintaining their percolated network under tensile strain has proven challenging due to the limited length of the fabricated metal NWs (1–20 μm).^[^ ^16^, ^45^, ^70^, ^71^ ^]^ In 2013, Lee et al. successfully demonstrated fabrication of long AgNWs over 500 μm via a multistep growth method using repetitive AgNO_3_ reduction in ethylene glycol solution with poly(vinyl pyrrolidone).^[^ ^72^ ^]^ Each AgNO_3_ reduction step accumulates additional AgNW segments onto the existing AgNWs while maintaining their diameter. The resulting long AgNWs were then integrated into an Ecoflex substrate, forming a network that exhibited 50% stretchability without resistivity change. Furthermore, by applying 300% pre‐strain to the Ecoflex substrate, the AgNW network could be stretched up to 460% strain, demonstrating its remarkable stretchability.

The remarkable conductivity and stretchability of AgNW networks have facilitated the development of intrinsically stretchable light‐emitting devices.^[^ ^41^, ^45^, ^73^, ^74^, ^75^, ^76^, ^77^, ^78^, ^79^, ^80^ ^]^ Liang et al. employed AgNWs with an aspect ratio of approximately 500 (10–20 μm in length and 25–35 nm in diameter) to fabricate intrinsically stretchable PLED arrays.^[^ ^45^ ^]^ The AgNWs were embedded in a flexible poly(urethane acrylate) (PUA) matrix, and the as‐fabricated AgNW–PUA electrode exhibited electrical conductivity under more than 100% strain. Although its sheet resistance changed from 15 to 65 Ω sq^−1^, the AgNW–PUA electrode survived 1500 cycles of stretching and relaxing with a peak strain of 30%. Furthermore, the AgNW–PUA electrode exhibited a transmittance of over 81% in the 500–1000 nm wavelength range, making it suitable for light‐emitting devices. As a proof of concept, Liang et al. demonstrated a polymer light‐emitting electrochemical cell (PLEC) and a 5 × 5 PLED pixel array using the AgNW–PUA electrodes. Both devices were intrinsically stretchable, and the PLEC was capable of being stretched up to 120%. The same research team extended their investigation by enhancing the stability of AgNW–PUA electrodes through GO soldering.^[^ ^41^ ^]^ As shown in Figure 2b, Liang et al. wrapped the AgNWs and their junctions with GO sheets to improve both electrical and mechanical stabilities. The GO–AgNW network exhibited enhanced repeatability in stretching, which can be attributed to the soldering effect of the GO on the AgNW junctions. This soldering mechanism effectively prevented the disjointing or sliding of the NWs during deformation. As a result, the integration of GO–AgNW–PUA electrodes enabled the realization of PLEDs with an enhanced intrinsic stretchability of 130% (Figure 2b). AgNW networks have also been utilized in the development of stretchable ACEL devices.^[^ ^75^, ^76^, ^77^, ^79^, ^80^ ^]^ The continuous advancement and commercial availability of AgNW technologies have led to the optimization of AgNW‐embedded polymer electrodes, resulting in significantly reduced sheet resistance, as low as few Ω sq^−1^. This enables ACEL devices to easily achieve a stretchability of 100% while maintaining an optical transmittance of over 80%. As a result, AgNW‐based electrodes have also been widely employed in the fabrication of stretchable electroluminescent fibers^[^ ^75^, ^76^ ^]^ and have found utility in recent research on stretchable display devices.^[^ ^47^, ^48^, ^78^, ^81^, ^82^ ^]^

While AgNWs have been commonly used as 1D metallic nanomaterials for intrinsically stretchable displays, there has been a growing effort to explore the utilization of CuNWs due to their potential cost efficiency.^[^ ^20^, ^83^, ^84^ ^]^ Tran et al. successfully fabricated super long CuNWs, ranging from 200 to 500 μm, with an impressive aspect ratio exceeding 3000.^[^ ^83^ ^]^ The resulting CuNW network, integrated onto a PDMS substrate, exhibited sheet resistances ranging from 6.9 to 90 Ω sq^−1^ and transmittance values ranging from 41% to 91% at a wavelength of 550 nm, depending on the density of the NWs. By employing a CuNW–PDMS electrode with a sheet resistance of 60 Ω sq^−1^ and transmittance of 86.7% as the top electrode, a 100% intrinsically stretchable ACEL device with bending, rolling, and twisting capabilities was demonstrated.

The 1D nanofibrous materials have also been utilized as stretchable light‐emitting layer.^[^ ^42^, ^44^, ^48^, ^81^, ^82^ ^]^ In many cases, intrinsically stretchable light‐emitting devices were limited by the stretchability of their emissive layers. To address this issue, Kim et al. introduced a small‐molecule nonionic surfactant, Triton X, as a plasticizer to enhance the stretchability of the commercially available emissive material, “Super Yellow (SY)”.^[^ ^48^ ^]^ By incorporating the plasticizer, the interchain interaction of the SY polymer chains was reduced, resulting in an increase in free volume, the formation of a 1D nanofibrous network, and an increase in softness. The Young's modulus of SY decreased from 412 to 77 MPa, while maintaining its carrier mobility, and the crack onset strain (COS) increased from 40% to 80%. A similar effect was observed when Triton X was added to PEDOT:PSS, a commonly used hole‐transport layer material. The Young's modulus of the hole‐transport PEDOT:PSS layer was decreased from 100 to 2.5 MPa, and its COS was increased from <10% to >160%. By incorporating the Triton‐X‐mixed SY emissive and PEDOT:PSS hole‐transport layers, an intrinsically stretchable OLED was demonstrated. At 80% of strain, the OLED maintained 50% of its unstretched luminescence value, reaching a peak luminance of 1167 cd m^−^^2^ at 12 V. This performance was maintained even after subjecting the device to 200 cycles of 40% strain. In addition, Zhang et al. optimized the SY to PU ratio to reduce the aggregation of SY nanofibers within the emissive layer.^[^ ^44^ ^]^ This optimization resulted in a uniform distribution of SY nanofibers in the PU matrix. The modulus of the SY–PU layer decreased from 4.9 GPa to 205 MPa depending on the PU wt%, while the crack formation strain increased up to 500%. An all‐polymer LED based on this SY–PU emissive layer exhibited a brightness of up to 7450 cd m^−2^ at 15 V, with a maximum current efficiency of 5.2 cd A^−1^ and approximately 100% stretchability.

### 2.3 0D Micro/Nanoparticles and QDs for Intrinsically Stretchable Displays

**Figure 3:**
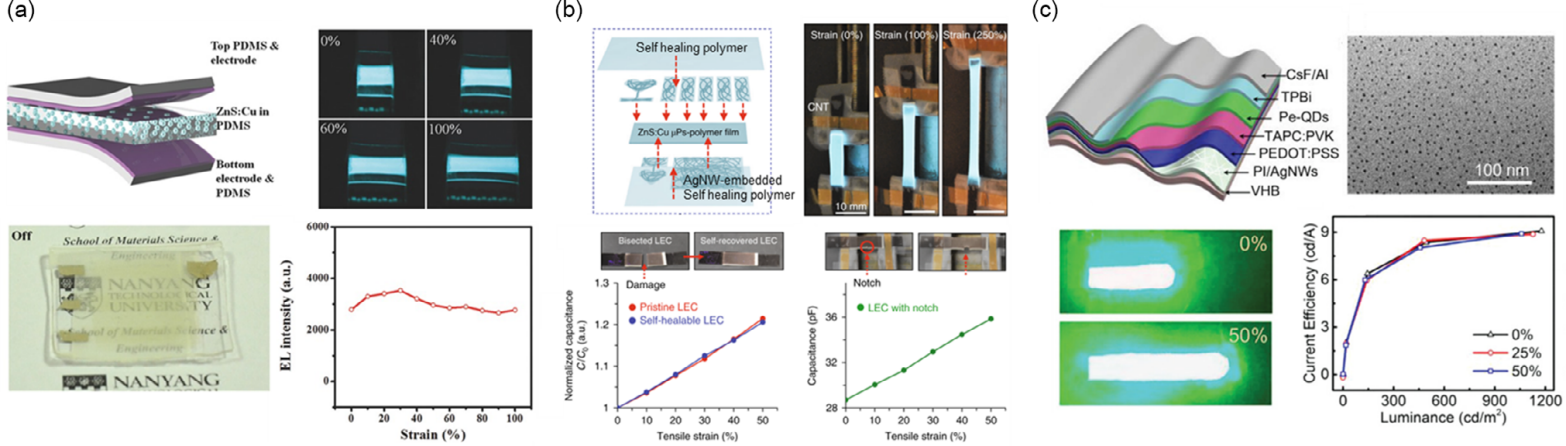
Schematic illustrations of intrinsically stretchable displays enabled by 0D particles and quantum dots (QDs). a) Stretchable ACEL device with ZnS:Cu–poly(dimethylsiloxane) (PDMS) light emissive layer. Simple ZnS:Cu microparticles–PDMS composites enabled high stretchability and robust light intensity up to 100% stain. Reproduced with permission.^[^ ^80^ ^]^ Copyright 2015, Wiley‐VCH. b) Self‐healable and intrinsically stretchable light‐emitting capacitor (LEC) device with ZnS:Cu light emissive layer. The device maintains its characteristics after cutting and healing. Reproduced with permission.^[^ ^87^ ^]^ Copyright 2018, The Authors, published by Springer Nature. c) Structure of the perovskite QD LED (QLED) and transmission electron microscopy (TEM) image of the MAPbBr_3_ QDs. Optical images and current efficiency of the LED at different strains are also displayed. Reproduced with permission.^[^ ^90^ ^]^ Copyright 2019, Wiley‐VCH.

In the context of stretchable light emissive layers, 0D micro/nanoparticles (NPs) and QDs have found significant utility. Specifically, for ACEL or light‐emitting capacitor (LEC) devices, a commonly employed approach involves blending ZnS:Cu phosphor microparticles into stretchable elastomers, thus forming stretchable light emissive layers.^[^ ^43^, ^65^, ^66^, ^67^, ^80^, ^85^, ^86^ ^]^ ZnS:Cu phosphor is a well‐studied material for ACEL devices and is readily available commercially. An example of the application of ZnS:Cu phosphor in stretchable emissive layers can be seen in the work of Wang et al.^[^ ^80^ ^]^ As illustrated in the schematic diagram of **Figure** **3**a, they prepared a mixture of ZnS:Cu phosphor and PDMS to establish a stretchable emissive layer. They also employed AgNW networks on PDMS as both the top and bottom electrodes. The resulting ACEL device, consisting of AgNW–PDMS/ZnS:Cu–PDMS/AgNW–PDMS layers, demonstrated well‐preserved luminous intensity even under 100% tensile strain (Figure 3a). Due to the simplicity of the device configuration, ACEL or LEC devices can be easily stretched up to a couple of hundreds of percentage of strain,^[^ ^65^, ^85^ ^]^ and the activation mechanism of the ZnS‐based phosphor through alternating current further facilitates the development of both self‐healing and stretchable display devices.^[^ ^65^ ^]^ The self‐healing capability provides reliability and long‐term stability to the devices in various practical situations. Shi et al. developed a self‐healable and stretchable ACEL device with a ZnS phosphor layer sandwiched between two aligned CNT‐based electrodes. The self‐healing PU matrix enabled the recovery of the devices after being cut. By healing the device at 60 °C, the device regained over 98% of its original luminance under 0%–400% strain and after 350 cycles of a fatigue test. ZnS:Cu phosphor has also been utilized in developing a self‐healing and stretchable LEC for an electronic skin system. Son et al. demonstrated a self‐healable and stretchable LEC array by embedding ZnS:Cu particles into self‐healable polymer matrix formed by incorporating a cross‐linked network of 4,4′‐methylenebis(phenyl urea) unit (MPU) and isophorone bisurea unit (IU) into a PDMS backbone (PDMS–MPU_0.4_–IU_0.6_).^[^ ^87^ ^]^ With CNT‐ or AgNW‐based self‐healable electrodes, the LEC exhibited high stretchability up to 250% and excellent healing capability even at room temperature by recovering its electrical characteristics after damage (Figure 3b). The self‐healable and stretchable LEC was further integrated into an electronic skin system with a self‐healable and stretchable strain sensor and electrocardiogram sensor.

In the case of stretchable PLEDs and OLEDs, which face challenges such as relatively high turn‐on voltage and low luminescence, the integration of inorganic QDs has been introduced as a possible solution.^[^ ^24^, ^88^, ^89^ ^]^ QDs are nanoscale semiconductor particles that emit light at specific wavelengths determined by their size, shape, and composition, offering a wide range of color possibilities. Their high luminescence properties make them attractive for enhancing the brightness of stretchable displays. Another notable advantage of QDs is their low operating voltage, which can contribute to energy efficiency in stretchable displays. Although QD‐based intrinsically stretchable display technologies are still immature, there have been several studies demonstrating their potential.^[^ ^88^, ^90^, ^91^ ^]^ Bade et al. embedded organometal halide perovskite microcrystals, methylammonium lead tribromide (MAPbBr_3_), in poly(ethylene oxide) (PEO) to develop stretchable emissive layers for LEDs.^[^ ^88^ ^]^ PEO provided mechanical stability to the brittle MAPbBr_3_ crystals under deformation. Intrinsically stretchable LEDs were then developed with this perovskite–PEO emissive layer and exhibited a 2.4 V of turn on voltage and a brightness of 15 960 cd m^−^^2^ at 8.5 V (approximately 2.5 cd A^−1^ of efficiency). These values were well maintained up to 40% strain. In another study, Li et al. reported the use of MAPbBr_3_ organometal halide perovskites with dimensions comparable to QDs, featuring an average diameter of 7 nm (Figure 3c).^[^ ^90^ ^]^ To fabricate free‐form devices with the perovskite QDs, an AgNW network and a PEDOT:PSS‐based transport layer were coated on an ultrathin (2 μm) PI film. Subsequently, nano‐thin perovskite QD light emissive layer, electron‐transport layer, and cathode were stacked to form an ultrathin QD LED (QLED) as illustrated in Figure 3c. Although the stretchable QLED demonstration with these perovskite QDs included structural modification via pre‐strain, the device showcased an impressive luminescent efficiency of up to 9.2 cd A^−1^ and a low 3.2 V turn on voltage, further highlighting the potential of QD‐like perovskite materials for stretchable displays.

### 2.4 Using Different Dimensions of Materials for Intrinsically Stretchable Displays

As highlighted in the preceding sections, nanomaterials with diverse dimensions play distinct roles and present varied opportunities in achieving intrinsically stretchable displays. Emissive layers, for instance, commonly employ 0D or 1D materials embedded in thin elastomers. In contrast, electrode materials often feature a 1D conductive network or a 1D network enhanced by a 2D functional sheet. Consequently, most of the recently reported works incorporate nanomaterials from multiple dimensions. For instance, in one of the state‐of‐the‐art studies by Zhang et al., all device layers were fabricated as nanoscale 2D films, except for the encapsulation layers, and 1D fibrous materials were embedded for the emissive layer.^[^ ^44^ ^]^ In another case, Chang et al. demonstrated a self‐powered, self‐healing, and intrinsically stretchable ACEL device by utilizing 1D SWCNT networks for stretchable electrodes and embedding 0D ZnS:Cu particles into an elastomer for the light emissive layer.^[^ ^66^ ^]^ As intrinsically stretchable displays have progressed through the integration of various nanomaterials, the ongoing advancement and optimization of these nanomaterials for their specific roles in intrinsically stretchable displays hold great promise for the realization and commercialization of stretchable displays.

## 3 Structurally Stretchable Displays

In the previous section, we discussed the role of nanomaterials in enabling intrinsically stretchable displays. In this section, we will next delve into design strategies and recent advancements in structurally stretchable displays and how nanomaterials have catalyzed their evolution. Geometric or structural design offers a universal approach, providing a potential pathway for realizing commercially viable free‐form displays without the challenges inherent to intrinsically stretchable displays.^[^ ^22^, ^92^, ^93^ ^]^ Structural design approaches allow engineers to relocate strain, reducing stress concentration at key regions such as hinges and interfaces and even alleviating strain in general. A major premise of structural design is to achieve stretchability beyond the intrinsic material limits, as moderate strains can lead to crack formation, degradation in structural strength, and loss of conduction paths.^[^ ^1^, ^94^ ^]^ Particularly, a primary design objective for structurally stretchable displays is the reduction of thickness of the material layers, where nanomaterials with small sizes and dimension provide fundamental advantages, such as reducing mechanical stiffness. By minimizing and evenly distributing stretching‐induced stress to mitigate fatigue failure, this approach enables the reversible transition between conventional planar 2D and sophisticated 3D display form factors.

To adapt free‐form displays to existing applications or replace current technologies, the resolution or pixel density should be maintained under deformation and match that of conventional displays, ideally greater than 200 pixels per inch (PPI).^[^ ^10^, ^95^, ^96^ ^]^ Additionally, structural designs should not compromise high efficiency, brightness, or low‐power operation, especially for wearable displays that require careful thermal management when attached to the skin or body.^[^ ^97^, ^98^ ^]^ Correspondingly, metamaterials or stretchable architectures leveraging kirigami and/or origami techniques effectively enhance heat dissipation (e.g., owing to high device voltage) to minimize user discomfort and ultimately achieve safer epidermal electronics.^[^ ^16^, ^99^, ^100^ ^]^ Optimizing the thickness of the encapsulation layer is also a critical consideration, as it involves a trade‐off between mechanical deformability and protection against moisture and oxygen permeation, which OLEDs are particularly sensitive to. Proper encapsulation systems require low modulus and high optical transmittance to not restrain device deformability and light emission efficiency, respectively. Nevertheless, the development of advanced encapsulation strategies beyond existing elastomeric substrates awaits stretchability imparted within the electroluminescent device layers.

There are three quintessential design strategies to structurally enable free‐form displays. The first approach involves buckled structures to accommodate stretching through out‐of‐plane deformation. The active and electrode layers are deposited on pre‐strained elastomeric substrates and the strain is then released, creating a wrinkled device that can flatten under elongation. Although this approach offers simple fabrication, it currently exists primarily at the research stage due to challenges in modulating surface wrinkles and achieving uniformity.^[^ ^101^, ^102^, ^103^ ^]^ The second approach is the island–bridge design, comprising stretchable bridges connected to rigid islands. The display layers are placed on the rigid and hence strain‐free islands, with any applied global strain transferred to the stretchable bridge regions, often with serpentine structures that act as springs.^[^ ^10^, ^104^ ^]^ This design is advantageous as it can be applied with existing non‐stretchable materials and fabrication techniques and is therefore closer to commercial application. However, this approach may entail lower pixel density due to in‐plane deformation and because the stretchable bridge regions have no electroluminescence.^[^ ^23^, ^105^ ^]^ As a related design to the island–bridge designs, the auxetic kirigami and origami strategies can impart structural stretchability without needing sizeable bridge structures. Both the first and second design approaches raise image distortion challenges due to the wavy surface morphology of buckled structures and the dispersed emissive units of the island–bridge structures. The third approach involves textile displays based on interlaced fibrous structures resembling clothing that can be worn on and conform to the users’ bodies. This approach is currently limited in its research and integration with nanomaterials. Additionally, development of stretchable textile displays depends heavily on the research of electroluminescent fibers, especially for the fiber‐based approach.^[^ ^106^, ^107^, ^108^, ^109^, ^110^ ^]^ A hybrid tessellation design approach with embedded planar pixel units on the textile surface has also been explored, though it tends to be porous, uneven, and/or rough due to the weaving structure.^[^ ^111^, ^112^, ^113^, ^114^ ^]^

### 3.1 Buckled Structures for Structurally Stretchable Displays

**Figure 4:**
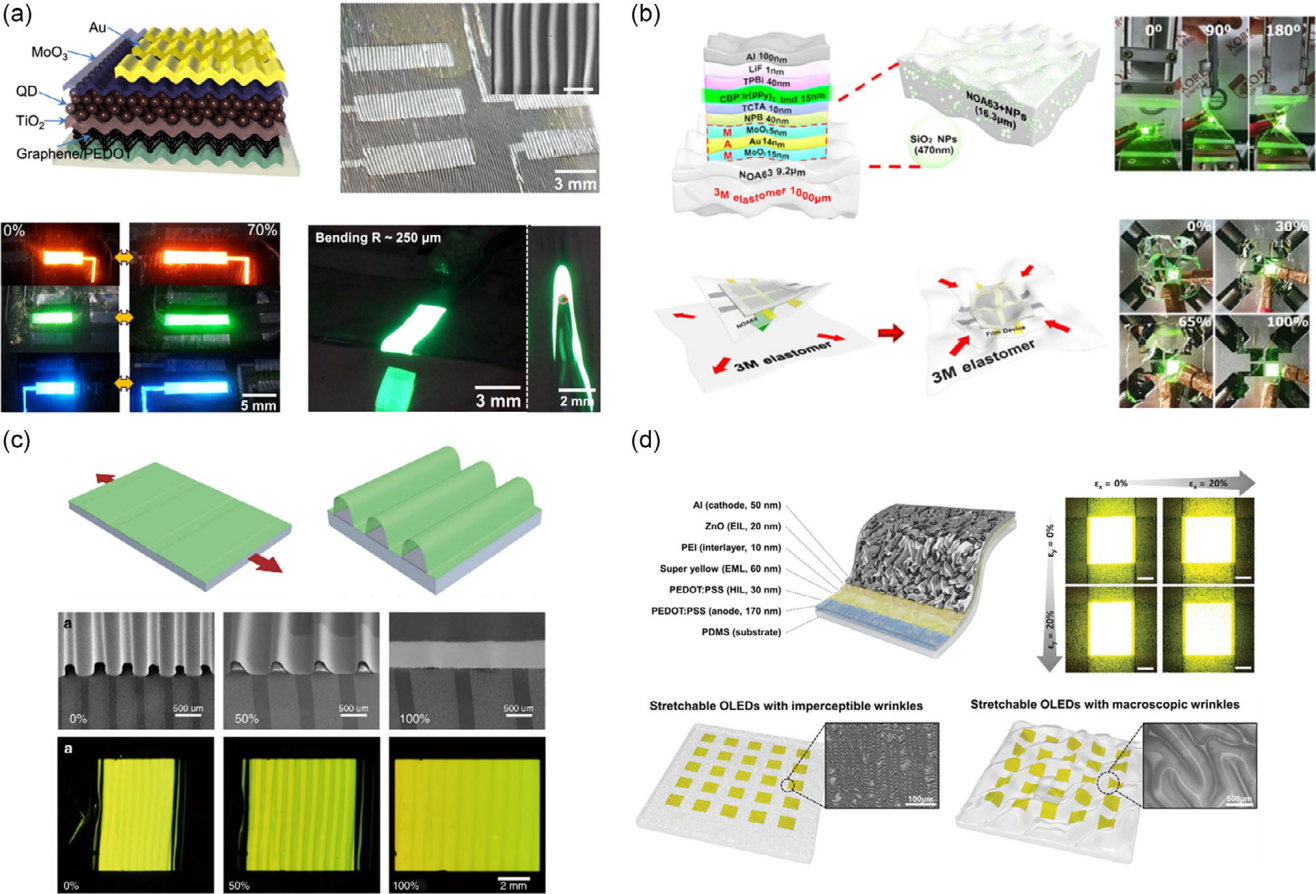
Schematic illustrations of stretchable display designs enabled by buckling structures and demonstrations of their stretchability. a) Wrinkled QLED based on colloidal QDs and graphene that can sustain up to 70% strain and 250 μm bending radius without performance degradation. Reproduced with permission.^[^ ^115^ ^]^ Copyright 2017, American Chemical Society. b) Geometrically stretchable OLED that can be twisted up to 180° and crumpled via biaxial strain. Reproduced under the terms of the CC‐BY Creative Commons Attribution 4.0 International license (https://creativecommons.org/licenses/by/4.0).^[^ ^97^ ^]^ Copyright 2021, The Authors, published by Springer Nature. c) Stretchable OLED with uniform periodic buckles fabricated by stencil‐pattern transferring technique. Reproduced under the terms of the CC‐BY Creative Commons Attribution 4.0 International license (https://creativecommons.org/licenses/by/4.0).^[^ ^103^ ^]^ Copyright 2018, The Authors, published by Springer Nature. d) Microwrinkled OLED with imperceptible wrinkles under 20 μm to produce distortion‐free pixel under 20% biaxial strain. Reproduced with permission.^[^ ^102^ ^]^ Copyright 2020, Wiley‐VCH.

The ultrathin thickness and robust mechanical flexibility of nanomaterials facilitate the design of buckled structures with device‐level stretchability while maintaining material integrity. Buckled structures are formed by transferring deposited planar device layers onto a pre‐strained elastomeric substrate and releasing the pre‐strain. The released pre‐strain then compresses and buckles the device layers, generating periodic wrinkles perpendicular to the strain direction. The degree of stretchability is directly proportional to the pre‐strain of the substrate. While inorganic LEDs (ILEDs) are generally rigid and difficult to engineer with buckled structures, OLEDs and QLEDs with thicknesses in the nanometer range and intrinsic softness have been actively used for the buckling strategy. Yin et al. reported a stretchable OLED, where the implementation of ultrathin and flexible device layers with a thickness of 200 nm allowed for biaxial stretchability imparted by biaxial pre‐strain‐induced buckling.^[^ ^33^ ^]^ The device demonstrated a maximum 50% tensile strain and current efficiency of 79 cd A^−1^. Kim et al. reported an electroluminescent device based on colloidal QDs (layer thickness of 260 nm) with enhanced stretchability via wrinkled structures with a monolayer graphene electrode (**Figure** **4**a).^[^ ^115^ ^]^ The excellent electronic properties and optical transparency of monolayer graphene significantly reduced the sheet resistance of the 50 nm‐thick PEDOT:PSS layer from 180 to 78.3 Ω sq^−1^ without compromising its optical transmittance. This enabled the graphene/PEDOT:PSS stack to be employed as the anode and hole‐injection layers of the stretchable QLED with a nanometer‐range thickness. The QLED device can be stretched up to 70% strain without performance degradation and folded with a 35 μm bending radius of curvature. In addition to imparting extreme flexibility, the transparent graphene electrodes provided low sheet resistance and reduced the operating and turn‐on voltages of the LEDs. The high conductivity of the graphene and QD layers also increased charge recombination probability, leading to high luminance despite relatively low current density versus ITO‐based LEDs.

In many cases, due to local microscopic buckling (typically at several tens of μm), placing the emissive layer near the neutral plane is crucial for mitigating the induced bending strain when designing buckled structures to ensure minimal performance degradation under strain. Kaltenbrunner et al. reported a compliant and imperceptible TFT by implementing the neutral plane design approach.^[^ ^116^ ^]^ They reduced the device size to sub‐micrometer level to significantly lower the flexural rigidity to achieve 233% stretching strain at the device level. In general, the elastomeric substrate is the thickest structural component and consequently dictates the neutral plane position. Accordingly, the parylene encapsulation layer was adjusted to be 0.8–1 μm thick to position the active layer close to the neutral plane. Correspondingly, tailoring the substrate material modulus or thickness can effectively tune the location of the neutral plane. Choi et al. developed a geometrically stretchable OLED under 2D stretching by thinning the substrate layer to position the rigid emissive layers at the neutral plane (Figure 4b).^[^ ^97^ ^]^ Norland optical adhesive 63 (NOA63) was employed as an encapsulation layer due to its excellent mechanical stability and optimal adhesion to the electrode layer owing to its hydrophilicity. The NOA63 encapsulation layer demonstrated lower stretchability due to its high Young's modulus of 1.5 GPa compared with PDMS (2 MPa) and PU (280 MPa), but the adoption of a thin 9.2 μm NOA63 layer effectively lowered the Young's modulus to 1.4 MPa. The resulting buckling‐enabled stretchable structure consisted of a 240 nm device layer, which included a 15 nm emissive 4,4′‐bis(N‐carbazolyl)biphenyl doped with 8% Ir(ppy)_3_ and a ductile and transparent 34 nm MoO_3_/Au/MoO_3_ electrode. The thin encapsulation was embedded with 470 nm SiO_2_ NPs to facilitate confined light. The device was capable of up to 100% applied strain with high current efficiency of 82.4 cd A^−1^ and external quantum efficiency of 22.3% and without efficiency roll‐off or high color stability (i.e., shifts in color coordinates). However, all these aforementioned buckled devices were inherently limited by the uncontrolled buckling morphologies, which impair device robustness and durability.

Achieving reliable control of the locally buckled structure (i.e., wrinkles) and ensuring surface uniformity at the device level are crucial for evenly distributing strain in the buckled state and maintaining image quality. However, the generation of periodic surface wrinkles remains the predominant challenge for the buckling design strategy. One method for improving control of buckle morphology is to improve the conformity between the device active layer and the elastomeric substrate. Ko et al. investigated 10–20 nm‐thick Ag electrodes capable of enduring 20% strain due to the wavy pattern induced by the pre‐strained PDMS substrate.^[^ ^117^ ^]^ The sub‐20 nm‐thick Ag exhibited optimal stretchability, and the smooth buckling conformal to the substrate enabled negligible electrical resistance change of the electrode upon extreme elongation. Additionally, the optical transmittance of the electrode could be tuned by the applied strain. The adhesion of the active layers to the elastomeric substrate is essential toward producing fine and periodic microwrinkle patterns without delamination during the pre‐strain release. Kim et al. studied solvent‐assisted pre‐strain methods for generating conformal periodic wrinkling by increasing the bond strength between the AgNW electrode network and the PDMS substrate.^[^ ^118^ ^]^ The rearrangement of AgNW networks due to solvent annealing prevented surface instabilities such as out‐of‐plane buckle delamination and formation of cracks and fractures due to modulus mismatch. The controllable electrode–substrate interface interaction led to uniform load transfer with minimal local stress concentration. As a result, the AgNW electrodes can be subjected to more than 50% strain and exhibited enhanced electromechanical stability with a high transmittance of 91.8% and a low sheet resistance of 26.8 Ω sq^−1^. As an alternative approach, Yin et al. showcased a laser‐programmable uniaxial buckling process to generate ordered periodic buckling profiles for stretchable displays.^[^ ^119^ ^]^ A femtosecond laser was used to generate 1D periodic gratings on an elastomeric substrate, where the resulting grooves suspended the buckled device layer. An OLED layer with 199 nm thickness was fabricated, and the device exhibited 70 cd A^−1^ efficiency at 70% strain. The device also endured extreme tensile strain of 100%, with only minor fluctuations in performance observed over 15 000 stretching cycles. Subsequently, Yin et al. extended their research and reported the use of a stencil‐pattern transfer technique to fabricate wrinkled structures with an ordered buckling profile (Figure 4c).^[^ ^103^ ^]^ To reduce the complexity of the fabrication process, Chen et al. presented a transfer‐free technique for the formation of ordered wrinkles with a period of 70 μm on a 204 nm OLED layer, achieving 20% stretchability while maintaining electroluminescent performance (current efficiency of 70.3 cd A^−1^) similar to rigid OLEDs on glass substrates.^[^ ^120^ ^]^ A 1 μm‐thick parylene encapsulation film evaporated via chemical vapor deposition was employed due to its low water vapor permeability, accessible room temperature deposition process, and controllable film thickness. In contrast to 20% luminance decrease for nonencapsulated device, the encapsulated device showed stable luminance after 10 h. Even after 2000 stretching cycles, 90% of the device luminance remained from its initial luminance of 760 cd m^−2^.

Another challenge for buckled structures is that they may suffer from light transmission reduction due to light scattering caused by the nonplanar structure when the device is relaxed. The reduction of wrinkle wavelengths to realize microwrinkled structures helps to minimize image distortion and enhance the display quality. In general, thinner films allow for shorter wavelengths of buckling.^[^ ^121^, ^122^ ^]^ However, fabricating the device on a plastic film and then transferring it to a pre‐strained elastomer limits the shortening of the buckle wavelength due to the micrometer‐scale thickness of the plastic film. To address this limitation, Jeong et al. introduced a direct deposition of PLEDs onto a pre‐strained PDMS substrate, effectively reducing the thickness of the wrinkled film down to 340 nm to shorten the wrinkle wavelength.^[^ ^102^ ^]^ Due to the hydrophobicity of PDMS, a low‐temperature solution process was employed to facilitate the transfer printing process. With this technique, the wavelength of the periodic wrinkle of the PLEDs was significantly reduced from the macroscopic level (at several hundred micrometers) to under 20 μm, mitigating image distortion at the device level due to pixel deformation (Figure 4d). The resulting microwrinkled PLED structure exhibited imperceptible periodic wrinkle patterns, which were not recognizable with the naked eye, allowing for 20% biaxial strain while maintaining similar performance to the planar structure. The microwrinkled PLED achieved a luminance over 8000 cd m^−2^ and a current efficiency of 7.76 cd A^−1^.

In addition to this traditional buckling design approach, which widely employed nano‐thin materials, a novel crossover design has emerged that encompasses both intrinsic material engineering as well as structural designed stretchability. For instance, there are mesh and pattern‐layered designs that can be implemented to relieve stress concentration at the device layers. Park et al. introduced a Ti NP‐embedded indium zinc oxide (IZO) electrode, enhancing the stretchability of transparent electrodes for stretchable OLEDs.^[^ ^94^ ^]^ A 200 nm‐thick IZO mesh with 10 μm diameter holes spaced at 10 μm intervals was fabricated, which exhibited superior mechanical properties and strain tolerance compared to the IZO planar electrode. The open hole areas of the mesh also contributed to improved optical transmittance. Additionally, by incorporating 2 nm‐thick Ti NPs, they enhanced the electrical conductivity of the IZO mesh by filling the hole areas. The other device components were also fabricated as nano‐thin films, and the thermally activated delayed fluorescence blue OLED based on this NP‐micromesh electrode sustained its performance up to 100% strain with only 20% resistance change after 1000 stretching cycles. Moreover, surface corrugation resulting from the mesh structure reduced the waveguide mode at the interface and enhanced the outcoupling. Rigid active layers can also be integrated with the buckling design approach, where wrinkled regions function as interconnects while the active layer remains flat throughout the stretch–release cycle, conceptually similar to island–bridge structures.^[^ ^123^ ^]^

The enhancement in the modulation of buckling patterns can benefit from recent studies on mechanically guided assembly.^[^ ^124^, ^125^, ^126^ ^]^ Mechanically guided assembly is a fabrication technique that can transform planar 2D electronic devices into complex 3D structures with extensive material compatibility including multilayer structures, precise controllability of the final buckled patterns, and scalability of the process. Ahn et al. reported a covalent bonding‐based 2D nano‐transfer that enabled buckled 3D nanostructures with a period as small as 50 nm.^[^ ^125^ ^]^ Through the manipulation of nanopattern shapes, buckling directions, and buckling modes, various desired 3D structures were successfully demonstrated, indicating that the mechanically guided assembly technique is key to enhance the design of buckled morphology through producing buckling profiles that are highly uniform and imperceptible with nanoscale periods.

### 3.2 Island–Bridge, Auxetic Kirigami, and Origami Structures for Structurally Stretchable Displays

Island–bridge structures employ stretchable bridge structures to connect rigid main pixel units containing the active layers. The compliant interconnects elastically elongate to impart stretchability, allowing the island regions to remain strain‐free. Thus, while the buckling design strategy only works for active layers with low film thicknesses and stiffnesses, the island–bridge approach provides greater flexibility and can accommodate a wider range of materials. Stretchable serpentine bridge structures encompass various designs that include horseshoe, U‐shape, sinusoidal, zigzag, and square/rectangular patterns.^[^ ^23^, ^117^, ^127^, ^128^, ^129^ ^]^ These design strategies can be integrated with existing well‐established rigid materials layers (e.g., ILEDs that can typically undergo <1% strain) and mature fabrication processes to facilitate the research and development of free‐form displays with comparable performance to mainstream rigid devices.

**Figure 5:**
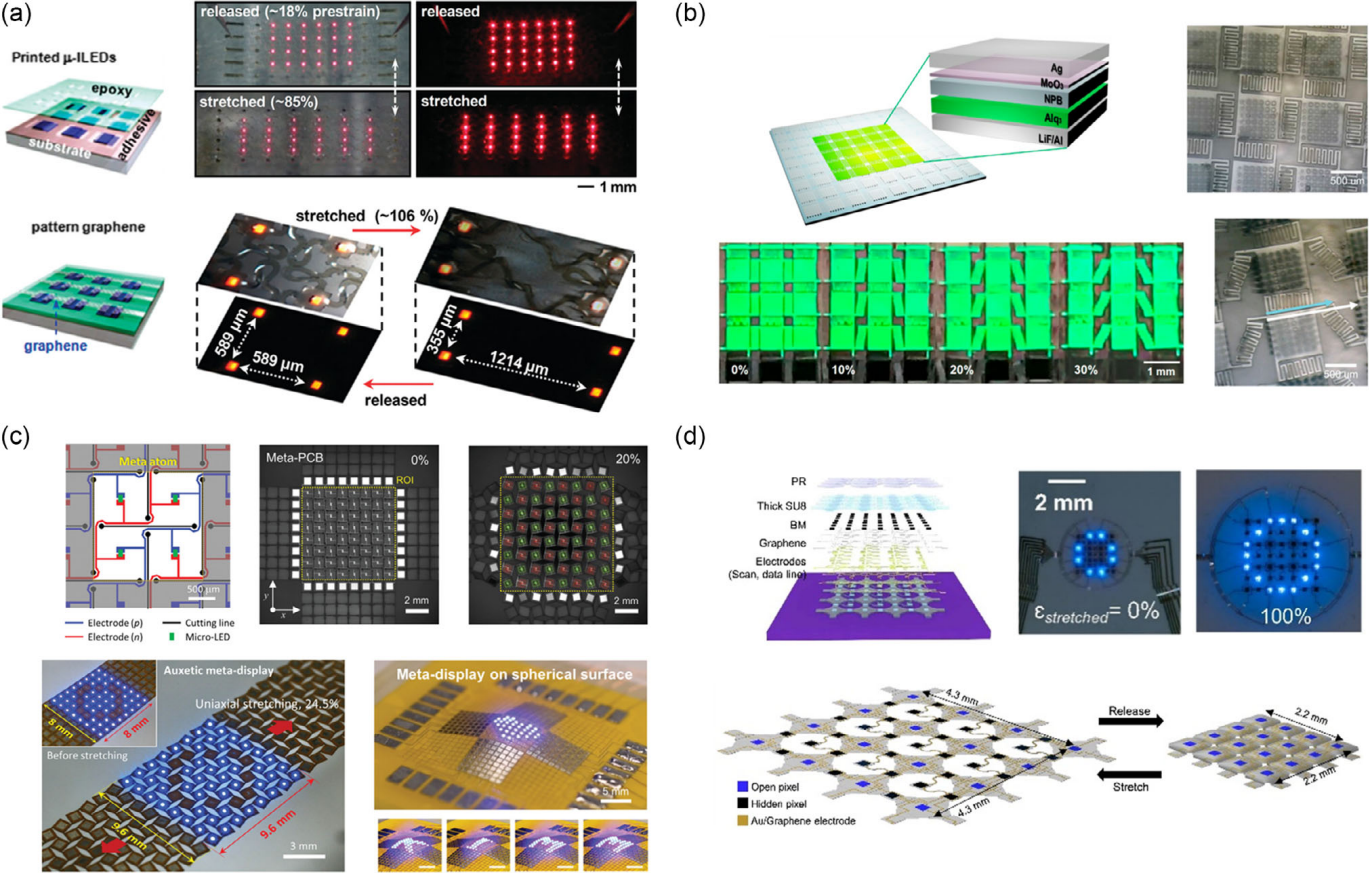
Schematic illustrations and demonstrations of representative stretchable displays based on island–bridge, kirigami, and origami structures. a) Stretchable LED with serpentine interconnects based on transparent monolayer graphene that can be stretched up to 106%. Reproduced with permission.^[^ ^131^ ^]^ Copyright 2011, American Chemical Society. b) Stretchable OLED via pillar arrays on a stress‐relief stretchable substrate to facilitate 30% strain. Reproduced with permission.^[^ ^92^ ^]^ Copyright 2020, American Chemical Society. c) Auxetic meta‐display with Poisson's ratio of −1 that can be subjected to 24.5% stretchability with no image distortion and attached to spherical surface with nonzero Gaussian curvature. Reproduced with permission.^[^ ^100^ ^]^ Copyright 2022, Wiley‐VCH. d) Morphable 3D display with hidden pixels to maintain pixel density due to stretching. Reproduced with permission.^[^ ^161^ ^]^ Copyright 2022, Elsevier.

When compared to buckling strategies, island–bridge strategies impart substantial stretchability with minimal added complexity due to the dedicated elastic interconnect structures. Kim et al. first developed an island–bridge structure based on arrays of GaAs micro‐LEDs with 2.5 μm‐thickness joined by metal serpentine interconnects.^[^ ^130^ ^]^ They demonstrated 75% axial stretching over 100 000 cycles without significant degradation in electrical properties. This unique island–bridge structural design foundationally established this novel design strategy for stretchable electroluminescent devices. Leveraging atomically thin nanomaterials to enhance stretchability, Kim et al. explored the use of monolayer graphene stacks via top‐down lamination for designing transparent interconnects to accommodate >100% strain (**Figure** **5**a).^[^ ^131^ ^]^ As a robust electrical interconnect, graphene provides high conformability to irregular surface topographies with exceptionally low flexural rigidity (2.31 × 10^−19^ N m).^[^ ^132^ ^]^ Any stretching‐ or bending‐induced stresses are concentrated at the serpentine bridge structures, and accordingly, the stretchability of the overall display device then primarily depends on the bridge structures while active layers at the island regions remain protected from the applied global strain.^[^ ^133^, ^134^ ^]^ To further improve stretchability, Kim et al. then implemented a hybrid platform consisting of serpentine bridges with a bilayer substrate, where the top layer with ultralow elastic modulus relieves stress while the bottom layer elastomeric substrate governs device stretchability. This was demonstrated in a stretchable OLED device capable of withstanding 140% strain despite the use of rigid materials as the active layer at the islands.^[^ ^135^ ^]^ The hybrid platform mitigated bending strain at the island regions to improve surface conformability (e.g., for wearable applications). However, this enhanced stretchability was achieved at the expense of increased device thickness due to the elastomeric layers. To preclude the substrate from constraining the overall device stretchability, Song et al. reported a cellular encapsulation strategy.^[^ ^136^ ^]^ This approach involved using stacked multilayer network materials to integrate inorganic electronic components. In comparison to conventional encapsulation methods, the cellular encapsulation using 50 μm‐thick PI network material patterned with periodic triangular lattices of microstructures enabled stretchability of up to 20% and high areal coverage of 110%. The contact between the network materials and the interconnects relied on weak van der Waals interactions, allowing serpentine interconnects to slide and deform freely.

Despite achieving superior stretchability over buckling structures, the island–bridge design strategy is uniquely constrained by the inherent trade‐off between pixel density and stretchability due to its primarily in‐plane elongation. The large gap between the electroluminescent pixel areas leads to lower areal coverage and image distortion when stretched. Small aperture (i.e., island‐to‐bridge area) ratio typically implies enhanced stretchability in the bridges due to their greater proportional surface area. Interconnects typically occupy around 50% of island–bridge device surface area to achieve 5% stretchability, and the aperture ratio decreases further under applied strain, creating image distortion.^[^ ^105^ ^]^ Nevertheless, the topology optimization of serpentine interconnects within the gap area such as by using fractal design to improve stretchability can overcome this challenge.^[^ ^128^, ^137^, ^138^, ^139^ ^]^ Lim et al. reported an island–bridge structure featuring compact serpentines with maximized coverage of gap spacing between adjacent islands, resulting in a nearly 90% aperture ratio in the unstretched state (Figure 5b).^[^ ^92^ ^]^ An intermediate pillar array structure between the island–bridge layer and the PDMS substrate enabled 35.3% stretchability without sacrificing pixel density. Furthermore, Nam et al. extended this work by designing a new serpentine connection to allow the rotation of islands to reduce the maximum local stress by one third.^[^ ^140^ ^]^ Alternatively, Lee et al. employed zigzag micro‐cracked bridge structures as opposed to their curvy counterparts to fabricate a stretchable organic optoelectronic system for health monitoring.^[^ ^141^ ^]^ The stretchable patch maintained stable performance while subjected to 30% strain via micro‐cracked Au interconnects with 50 nm thickness. Though the proof‐of‐concept device only exhibited 11 PPI, the design offered scalability up to 50–200 PPI via the reduction in pixel size. In a separate study, Oh et al. integrated rigid islands onto a fully stretchable interconnect substrate to reduce the size of gap regions and further improve display resolution.^[^ ^142^ ^]^ The pixel units were embedded directly onto the serpentine regions without requiring conventional rectangular island arrays with external bridge structures.

Alternatively, the positioning of stretchable interconnects into a separate layer beneath the rigid islands can further increase the aperture ratio. Multilayer island–bridge structures enable the reduction of gap area between pixels as the serpentine interconnects are tucked under the island areas. Accordingly, the modular design circumvents the inverse relationship between stretchability and display resolution for island–bridge structures. Extended beyond conventional single layer island–bridge structures, Biswas et al. presented a multilayer stretchable printed circuit board with integrated LEDs that enabled elongation up to 260%.^[^ ^143^ ^]^ Vertical integration between active layers on island and the metallic interconnects was achieved through vertical interconnect accesses with optimal electrical contact and stable mechanical contact between different layers. Kim et al. developed an island–bridge structure with a double‐layer modular design to increase the areal coverage to 77% with around 10% stretchability.^[^ ^105^ ^]^ The vertical integration of rigid ILEDs and the stretchable serpentine interconnects at a lower layer allowed stretchability to be independent of the areal coverage. The double‐layer design also allowed improved aperture ratio via mounting more ILEDs to increase the island surface area.

The contact and adhesion between the rigid island regions and the substrate represent an additional critical design criterion. The conformability of stretchable electronics has prompted studies into the adhesion of rigid layers to elastomeric substrates. Xiao et al. demonstrated a theoretical model to investigate the conformability of a relatively rigid island region attached to a torus surface.^[^ ^104^ ^]^ Utilizing the principle of energy minimization and material failure, a critical dimensionless parameter was formulated considering the island thickness, interfacial adhesion energy, and principal curvatures, thereby partitioning the conformal domain into distinct two regions. In the weak adhesion region, the maximum island strain remained below the failure strain during conformal contact, and delamination of the island would occur upon adhesion failure. Conversely, in the strong adhesion region, the island strain eventually surpassed the failure strain of the material layers and led to device failure. The modulus mismatch at the interface between the stiff island regions and the surrounding soft surface including the substrate and the interconnect induces high local stress concentration and potentially leads to premature material failure. Li et al. demonstrated the island strain effect through computational simulation studies and proposed an additional compliant buffer layer to relieve stress concentration.^[^ ^144^ ^]^ The buffer layer design strategy complementing island–bridge structures nearly doubled the stretchability. Lin and Tsai investigated interfacial fracture development between deposited island regions and stretchable film substrates at >200% applied tensile strain.^[^ ^145^ ^]^ The Ogden model was used to characterize the fracture energy between the stiff island regions and the thermoplastic PU substrate, which was subjected to nonlinear stretching behavior. The findings showed that the shearing mode dominated over the opening mode at the island–substrate interface. Moreover, the ratio of shear stress to normal stress slightly decreased and the interfacial fracture energy increased as the debonding length increased after the initial delamination.

Despite current limitations, the island–bridge design strategy represents the most readily accessible approach toward large‐scale stretchable displays. Choi et al. demonstrated the feasibility of stretchable active matrix (AM)‐ILEDs driven by Si–TFTs via roll‐based transfer printing with approximately 99.9% yield. This high yield was attributed to the accurate control of adhesion force (i.e., van der Waals interactions) between the roller stamp and target substrate.^[^ ^146^ ^]^ The display was capable of sustaining 40% tensile strain over 200 cycles owing to the strong adhesion between heterogenous material layers despite their elastic modulus mismatch. This printing technique can be implemented for layers with reduced film thickness and at a large scale due to the high overlay alignment. Consequently, transfer integration emerges as a practical approach for fabricating large‐area stretchable displays.^[^ ^147^ ^]^ More recently, Kim et al. fabricated a large 14.1 in. stretchable AM OLED display based an island–bridge structure with 300 cd m^−2^ that can withstand 10 000 stretch–release cycles though only with 5% stretchability.^[^ ^148^, ^149^ ^]^ Similarly, Jung et al. unveiled a 13 in. AM micro‐LED stretchable display enabled by an island–bridge design with 20% stretchability, 500 cd m^−2^ luminance, and 72 PPI resolution.^[^ ^150^ ^]^

Kirigami represents an alternative design strategy to produce discrete regions of rigid island areas with strategic incisions to enable stretchability without requiring external interconnect structures. The incisions serve as stress‐relief regions that shift strain away from the stiff island regions through out‐of‐plane deformation, occupying less surface area and thereby enabling greater resolution than conventional island–bridge designs with serpentine electrodes. Jang et al. developed a kirigami cutting strategy to fabricate an auxetic meta‐display that is stretchable without image distortion (Figure 5c).^[^ ^100^ ^]^ The display possessed an image resolution of 25–50 PPI and stretchability of 24.5% with a Poisson's ratio of −1, with isotropic scaling image transformation to preserve image quality. The auxetic metastructure imparted stretchability via incision regions that functioned as hinges, rotating the stiff square island regions to relieve stress when the device was stretched. The auxetic meta‐display was attached to a spherical surface that had nonzero Gaussian curvature without resulting in any image distortion and wrinkles/creases. However, the integration of kirigami design strategies with nanomaterials is still rudimentary toward the development of stretchable displays despite various kirigami‐enabled stretchable material designs for other applications.^[^ ^151^, ^152^, ^153^ ^]^ Rao et al. implemented kirigami designs on ultrathin optoelectronic pixel arrays of 1.25 μm thickness to enable curvy and shape‐adaptive imagers. For interconnection, Rao et al. deposited either a 5 nm Cu/100 nm Au or a 5 nm Cr/200 nm Au metal layer encapsulated by PI layers, resulting in an approximate total thickness of 3 μm. Owing to rotation induced at the metal interconnect regions to relieve strain without pixel deformation, the kirigami structure can endure up to 30% biaxial strain.^[^ ^154^ ^]^ In addition, kirigami design strategies have already been implemented toward fabricating highly stretchable electrode layers based on ultrathin nanomaterials.^[^ ^155^, ^156^, ^157^ ^]^ Yong et al. investigated surface conformal graphene stretchable electrodes based on kirigami architectures, demonstrating strain‐insensitive electrical properties under applied tensile strains of up to 240%.^[^ ^158^, ^159^ ^]^

As opposed to kirigami (i.e., cut paper) designs, origami (i.e., folded paper) designs can impart stretchability to displays without incisions employed by kirigami, which instead utilizes out‐of‐plane deformation through folding of creases that serve as hinges. Upon device stretching, stress is suppressed at the facets, which represent the island regions containing rigid active layers. Deng et al. investigated the design of Miura‐like origami tessellations for stretchable displays with 30% stretchability.^[^ ^160^ ^]^ Similar to the working principle of the buckling approach, the 20–30 μm‐thick crease patterns remained folded during the unstretched state of the device but became flattened upon stretching. As a result, folding of the microscopic crease regions at 400 μm wide led to bending‐induced curvature and consequently in‐plane strain. The 500 nm‐thick Cu electrode layer was located only about 5 μm away from the neutral plane, and thus 3.4% bending strain was applied to the electrode layer even under maximum folding, which is much less than the material's elongation failure strain.

The design of origami patterns also enables greater conformability to different curvatures via tessellation optimization. Therefore, origami provides enhanced target surface conformability due to a readily reconfigurable 3D structure at the expense of added design complexity. Lee et al. developed a stretchable display with a morphable 3D structure by leveraging origami techniques (Figure 5d).^[^ ^161^ ^]^ When their display was stretched at 100% strain, pixel density and image quality were maintained due to unfolding of the origami structure to expose hidden pixels that filled the gaps. The device was based on GaN‐based micro‐LEDs with a 2.44 μm‐thick active layer and an electrode layer consisting of both Au (<100 nm) and Bi‐Gr, where the graphene layer further improved the mechanical and electrical stability. The incorporation of atomically thin graphene decreased the strain transferred to Au at the hinges from 1.13% to 0.88% and with a lower resistance change of 18.3% vs 24.9% for the bare Au electrode.

### 3.3 Textile Structures for Structurally Stretchable Displays

Textile display systems include various forms of interlaced yarns or threads accomplished by weaving, knitting, and braiding techniques. A variety of textile configurations based on knitting or weaving techniques, such as weft and tricot warp knits and plain biaxial weave, have been investigated.^[^ ^162^, ^163^, ^164^ ^]^ The small gaps between adjacent fibers facilitate sliding to relieve stress and subsequently impart stretchability, which can be tuned by adjusting the gap dimensions. The fiber‐based textile display design implements cylindrical fibers to provide advanced deformability such as 3D bending and twisting, where the electroluminescent unit envelops the fiber strand.^[^ ^2^, ^165^, ^166^ ^]^ Correspondingly, discrete fiber units are woven together to assemble a textile display. The alternative fabric‐based strategy is less complicated and employs conventional planar active layers deposited on top of the interlaced nodes of a textile electrode substrate network to enable device stretchability.

**Figure 6:**
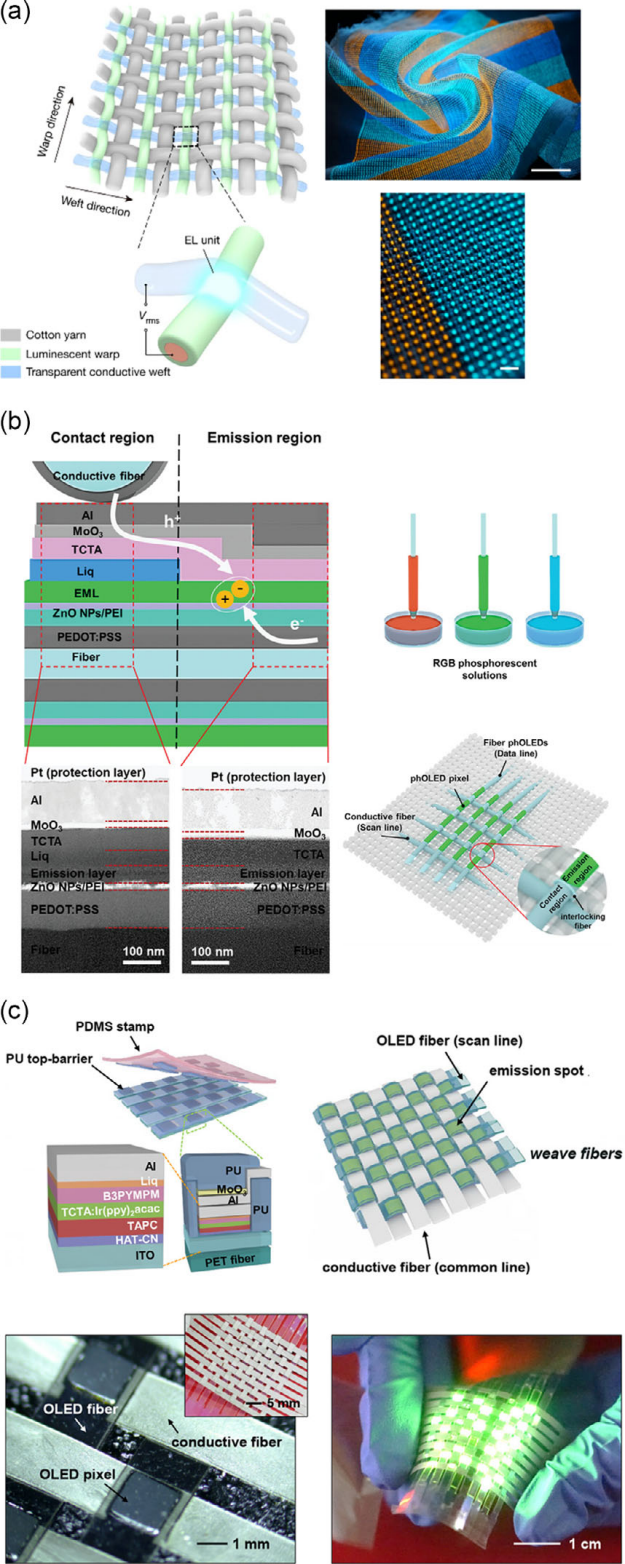
Schematic illustrations of stretchable displays leveraging textile structural design approach and their implementation. a) Large‐area fiber‐based textile display with multicolor OLED units at the weft–warp contact points, with breathable textiles capable of undergoing repeated machine washing. Reproduced with permission.^[^ ^167^ ^]^ Copyright 2021, The Authors, published by Springer Nature. b) Red, green, and blue (RGB) phosphorescent OLED based on a dip‐coating technique to uniformly coat cylinder‐shaped poly(ethylene terephthalate) (PET) fibers. Reproduced with permission.^[^ ^110^ ^]^ Copyright 2021, Wiley‐VCH. c) Fabric‐based textile display based on rectangular stripes with periodically patterned OLED pixels that can endure 20% stretchability. Reproduced with permission.^[^ ^113^ ^]^ Copyright 2020, American Chemical Society.

Fiber‐based textile displays are unique owing to the omnidirectional electroluminescence and deformability of the fiber strands compared to conventional planar devices. Shi et al. demonstrated a large‐area fiber‐based textile display by weaving conductive weft and luminescent warp fibers, where the weft–warp contact points constituted the micrometer‐scale electroluminescent units (**Figure** **6**a).^[^ ^167^ ^]^ The average luminance reported by Shi et al. was 122 cd m^−2^, which is comparable to commercially available rigid planar displays. The textile display also showcased <8% emission intensity degradation when subjected to bending, stretching, or pressing. The textile display could also endure 1 mm bending radius. The electric‐field‐driven ZnS phosphor emissive layer was intrinsically durable and necessitated only spatial contacts between wefts and warps for electroluminescent emission, which mitigated issues of subpar Ohmic contact.^[^ ^99^ ^]^ Because the fibers were woven, the density of electroluminescent units could be easily tuned by adjusting the weaving parameters to change the distance between the weft–warp contact points, with the narrowest spacing reported only about 800 μm.

Furthermore, nanomaterials provide a passageway to reduce the fiber strand thickness and augment the electroluminescent performance due to their novel optical, chemical, and electronic properties when compared to bulk materials. Li et al. demonstrated thermochromic fibers that were coated with a reduced GO electrode layer with excellent conductivity and electrothermal stability.^[^ ^106^, ^107^, ^108^, ^109^, ^110^ ^]^ The conductive fibers could sustain up to 300% elongation over 1000 heating or stretch–release cycles. Moreover, thin nanofilms and NPs have been exploited as electrodes, functional layers, and electroluminescent layers enveloping the cylindrical fiber substrate. With nano‐thin device layers and ZnO NPs, Hwang et al. investigated RGB phosphorescent OLED fibers fabricated through a combination of concentric dip‐coating and thermal evaporation method (Figure 6b).^[^ ^110^ ^]^ The 290 nm‐thick device layer (100 nm of cathode, 10 nm of light emissive layer, 80 nm of functional layers, and 100 nm of anode) was synthesized on a cylindrical PET fiber substrate with 300 μm diameter. The fibers were encapsulated by a 50 nm‐thick AL_2_O_3_ layer through thermal atomic layer deposition to protect against moisture and oxygen. The phosphorescent OLEDs exhibited optimal electroluminescent performance with 4462, 11 482, and 1199 cd m^−2^ and 16.3, 60.7, and 16.9 cd A^−1^ for red, green, and blue, respectively. Furthermore, the fibers could endure up to 1.75% tensile strain, and there was no observable optoelectronic performance degradation until after 1000 bending cycles. Overall, dip‐coating and extrusion represent prevalent approaches for large‐scale fabrication of electroluminescent fibers, but their process precision, yield, and durability still remain inferior compared to vacuum deposition.^[^ ^168^ ^]^

Compared with fiber‐based designs, fabric‐based textile displays are structurally less complex, as the planar active layers are fabricated directly on top of woven electrode substrates. Song et al. presented a hybrid fabric‐based textile display via a woven network of perpendicularly arranged interconnectable conductive fibers with planar OLED layers on top (Figure 6c).^[^ ^113^ ^]^ Instead of implementing cylindrical electroluminescent fibers, existing planar LED layers were integrated into an electrode network due to their superior brightness, efficiency, and robustness. The phosphorescence OLEDs, with 336 nm thickness, were periodically patterned onto rectangular fiber stripes. A textile display based on 10 × 10 electrode arrays was demonstrated with a 1 mm gap between adjacent fibers, and it showed a stretchability of 20% strain with current efficiency of ≈46 cd A^−1^. The implementation of nanomaterial coatings could further improve electrical conductivity and electroluminescent performance under high stretchability. The PU‐based passivation led to a much slower performance decay compared to the device without passivation at ≈8 h in ambient condition. Furthermore, the passivated device could function for ≈7 h by immersing it in deionized water, whereas the absence of passivation destroyed the device immediately. The durability could be further enhanced through an elaborate encapsulation system. Wu et al. reported a ultrasheer knitted fabric with a ≈100 nm conformal gold coating as a conductive semitransparent electrode.^[^ ^112^ ^]^ The stretchable fabric endured 200% strain while retaining high conductivity (3.6 ± 0.9 Ω sq^−1^). The electroless nickel‐immersion gold metallization technique was used to produce conformal gold coating on individual fibers (87% nylon and 13% spandex) while maintaining the softness and stretchability of the original textile. This provides a platform for developing fabric‐based textile displays beyond the primitive integration of planar device layers onto fabric substrates with rough surfaces (e.g., clothes) via an intermediary planarization layer.^[^ ^169^, ^170^ ^]^

Despite recent impressive progress toward electroluminescent textile displays, the current configurations with emissive layer thicknesses of ≈30 μm require downscaling in size to improve stretchability. In particular, nanomaterials can be incorporated for design optimization. For instance, electrically conductive and robust graphene could enable high‐performance and extremely flexible electrodes while further decreasing the fiber thickness.^[^ ^171^ ^]^ One inherent weakness of the textile design is the difficulty in reducing device thickness when compared to the other two primary design strategies for achieving high‐resolution displays. In contrast, textile‐enabled stretchable electroluminescent devices may be more suitable for smart clothing or wearable displays where the gaps between pixel units provide breathability to increase user comfort and ameliorate heat dissipation. Nevertheless, textile strategies require higher operating voltages and frequencies when compared to stretchable displays based on other planar layer design strategies. Fiber‐based electroluminescent devices especially have worse electrical and optical efficiency. The longevity of textile displays is limited by material deterioration due to local friction generated at the interlaced nodes caused by the movement of individual fibers within the textile network during stretching. The commercialization of wearable textile displays is further complicated by durability concerns, particularly the exposure to dust and humidity while enduring repeated stretch–release cycles, which requires advanced encapsulation system to provide protection without sacrificing stretchability and electroluminescent performance.

## 4 Conclusion and Outlook

The development of stretchable displays has been a focus of research and innovation in recent decades, driven by advancements in various technologies such as nanomaterials, stretchable elastomers, conjugated polymers, and nanoscale structuring techniques. These advancements have paved the way for the realization of stretchable displays with increasing resolutions and improved stretchabilities. In this review article, we explored the specific roles of different nanomaterials in various strategies employed to develop stretchable displays.

Thin organic and inorganic films have enabled the development of flexible devices due to their low bending rigidity. By further thinning these materials to the nanoscale range and positioning them at the neutral plane, ultraflexible devices with extremely small bending radii have been developed, allowing for structural modifications such as buckling to achieve stretchability. Nano‐thin conductive films have found widespread use as interconnect materials in structurally stretchable devices with island–bridge, kirigami, and origami structures. Atomically thin 2D materials, such as graphene, have been utilized to enhance the mechanical robustness and conductivity of electrode materials, as well as the chemical stability of both intrinsically and structurally stretchable devices. Additionally, 1D nanofibrous materials have played a crucial role, especially toward intrinsically stretchable displays, enabling the development of stretchable and transparent conductors by embedding 1D metal NW or CNT networks into elastomers to form highly stretchable light emissive and functional layers. While percolated networks of microparticles/NPs have been less utilized than other nanomaterials, they also have been employed for stretchable conductors. Moreover, emissive micro‐phosphor particles have found wide applications in stretchable ACEL devices. QDs for stretchable and free‐form displays are still in their infancy but have gained increasing attention from researchers as highly efficient emissive materials for stretchable displays.

Despite technological advancements, practical applications of both intrinsically and structurally stretchable displays still face challenges. Intrinsically stretchable strategies need to address variations in device performance under strain, while structurally stretchable strategies suffer from nonuniform surfaces, losing emissive area under strain, or complex fabrication processes depending on the structure. Nevertheless, the progress made in nanomaterials and their composites and fabrication techniques has narrowed the technical gaps associated with stretchable displays. Further advancements are anticipated to catalyze the development of high‐resolution, highly efficient, and highly stretchable displays in the near future. Stretchable displays capable of free‐form deformation have the capacity to revolutionize the landscape of electronic devices, enabling new functionalities and applications that were previously unimaginable. As research and development efforts continue, we can look forward to exciting innovations and practical implementations of stretchable displays in various fields. These include wearable electronics, biomedical devices, portable and expandable displays, interactive displays, and beyond. The transformative potential of stretchable displays opens up new possibilities for enhanced user experiences and technological advancements in a wide range of applications.

## Conflict of Interest

The authors declare no conflict of interest.
